# An *Entamoeba*-Specific Mitosomal Membrane Protein with Potential Association to the Golgi Apparatus

**DOI:** 10.3390/genes10050367

**Published:** 2019-05-13

**Authors:** Herbert J. Santos, Yuki Hanadate, Kenichiro Imai, Tomoyoshi Nozaki

**Affiliations:** 1 Department of Biomedical Chemistry, Graduate School of Medicine, The University of Tokyo, 7-3-1 Hongo, Bunkyo-ku, Tokyo 113-0033, Japan; hjsantos@m.u-tokyo.ac.jp; 2 Department of Parasitology, National Institute of Infectious Diseases, 1-23-1 Toyama, Shinjuku-ku, Tokyo 162-8640, Japan; yuki@nih.go.jp; 3 Graduate School of Life and Environmental Sciences, University of Tsukuba, 1-1-1 Tennodai, Tsukuba, Ibaraki 305-8572, Japan; 4 Molecular Profiling Research Center for Drug Discovery, National Institute of Advanced Industrial Science and Technology (AIST), 2-4-7 Aomi, Koto-ku, Tokyo 135-0064, Japan; kenichiro.imai@aist.go.jp; 5 Biotechnology Research Institute for Drug Discovery, National Institute of Advanced Industrial Science and Technology (AIST), 2-4-7 Aomi, Koto-ku, Tokyo 135-0064, Japan

**Keywords:** *Entamoeba histolytica*, mitosome, *Entamoeba*-specific transmembrane mitosomal protein of 30 kDa, secretory pathway calcium ATPase

## Abstract

The aerobic mitochondrion had undergone evolutionary diversification, most notable among lineages of anaerobic protists. *Entamoeba* is one of the genera of parasitic protozoans that lack canonical mitochondria, and instead possess mitochondrion-related organelles (MROs), specifically mitosomes. *Entamoeba* mitosomes exhibit functional reduction and divergence, most exemplified by the organelle’s inability to produce ATP and synthesize iron-sulfur cluster. Instead, this organelle is capable of sulfate activation, which has been linked to amoebic stage conversion. In order to understand other unique features and components of this MRO, we utilized an in silico prediction tool to screen transmembrane domain containing proteins in the mitosome proteome. Here, we characterize a novel lineage-specific mitosomal membrane protein, named *Entamoeba* transmembrane mitosomal protein of 30 kDa (ETMP30; EHI_172170), predicted to contain five transmembrane domains. Immunofluorescence analysis demonstrated colocalization of hemagglutinin (HA)-tagged ETMP30 with the mitosomal marker, adenosine-5’-phosphosulfate kinase. Mitosomal membrane localization was indicated by immunoelectron microscopy analysis, which was supported by carbonate fractionation assay. Transcriptional gene silencing successfully repressed RNA expression by 60%, and led to a defect in growth and partial elongation of mitosomes. Immunoprecipitation of ETMP30 from ETMP30-HA-expressing transformant using anti-HA antibody pulled down one interacting protein of 126 kDa. Protein sequencing by mass spectrometry revealed this protein as a cation-transporting P-type ATPase, previously reported to localize to vacuolar compartments/Golgi-like structures, hinting at a possible mitosome-vacuole/Golgi contact site.

## 1. Introduction

The mitochondrion is a vital organelle in the cell as it is involved in key metabolic processes such as ATP generation via oxidative phosphorylation, iron-sulfur cluster formation, the tricarboxylic acid cycle, β-oxidation, heme biosynthesis, phospholipid, vitamin, steroid and amino acid metabolism, calcium homeostasis, and programmed cell death [1,2]. However, certain eukaryotic organisms that inhabit anoxic or hypoxic environments, lack canonical mitochondria, and instead possess highly divergent and often degenerate mitochondrion-related organelles (MROs). Particularly, a few species of parasitic protists contain MROs, which perform diversified functions and mechanisms that contribute to their parasitic lifestyle [3]. One example is the mitosome of the anaerobic protozoan *Entamoeba histolytica*, the etiological agent of amoebiasis, which causes an estimated 100,000 deaths annually [4]. Amoebiasis is a disease characterized by diarrhea and dysentery, which could lead to extra-intestinal complications such as abscess formation in the liver, lungs, and brain. Apart from having components that are essential to the cell’s survival [5,6], the *E. histolytica* mitosome contains enzymes that carry out sulfate activation [7] which leads to the formation of cholesteryl sulfate, a molecule linked to stage conversion from the metabolically active trophozoite form to the infective cyst form [8]. Such features highlight the importance of mitosomes not only on parasite proliferation but also on disease transmission with its involvement in amoebic cell differentiation. 

Unlike the canonical aerobic mitochondria, the *E. histolytica* mitosomal membranes have minimal and mostly divergent components, including transport systems for proteins and metabolites [9,10]. Only a few mitosomal outer membrane proteins have been identified, such as the pore forming β-barrel component of the translocase of the outer membrane (TOM) complex, Tom40 [11] involved in mitochondrial protein import [12], the central component of the sorting and assembly machinery (SAM) complex, Sam50 [11], involved in the assembly of β-barrel proteins on the outer membrane [12], and a lineage-specific mitosomal β-barrel outer membrane protein of 30 kDa, MBOMP30 [6]. On the inner mitosomal membrane, only the sodium/sulfate transporter [7] and two proteins that belong to the mitochondrial carrier family (MCF), namely ATP-ADP carrier protein [13], and phosphate carrier [11], are the known channel proteins reported. In our previous works, we developed in silico tools to predict membrane proteins, including a specialized prediction pipeline for proteins possessing α-helical transmembrane domains [14], and successfully confirmed our predictions through supporting cell biological and biochemical evidences [6,15]. Using this predictor, we found additional novel lineage-specific mitosomal membrane protein candidates, namely EHI_170120, which was confirmed to be localized to the mitosomal membranes, and EHI_099350, which demonstrated dual localization to mitosomes and the ER [14]. Here, we report another *Entamoeba*-specific mitosomal membrane protein predicted to possess five transmembrane domains, which we name *Entamoeba* transmembrane mitosomal protein of 30 kDa (ETMP30). It appears to interact with a cation-transporting P-type ATPase (EHI_065670) previously reported to be localized to vacuolar compartments that are postulated to be Golgi-like structures [16] in *E. histolytica*.

## 2. Materials and Methods

### 2.1. In Silico Predictions and Analyses

For the prediction of *Entamoeba*-specific membrane proteins, we used a prediction pipeline developed in a previous study, which combined highly-sensitive homolog search methods (JackHMMER [17] and HHblits [18]) and a novel transmembrane domain prediction method [14]. To find ETMP30 homologs in other *Entamoeba* species, we performed sequence similarity searches against the genome and proteome sequences of *E. dispar*, *E. moshkovskii*, *E. invadens,* and *E. nuttalli* in the *Amoebozoa* resource database, AmoebaDB [19], using blastp and tblastx [20]. For the analysis of transmembrane regions, we used ΔG prediction for transmembrane insertion [21]. For structural similarity prediction and sequence repeat analysis, we used HHpred [22] and HHrepID [23], respectively.

### 2.2. Amoeba Cultivation, Plasmid Construction, and Amoeba Transfection

Axenic cultures of *Entamoeba histolytica* HM-1:IMSS cl6 [24] and G3 [25] were maintained in Diamond BI-S-33 medium [24]. Total RNA extraction from *E. histolytica* HM1:IMSS clone 6, followed by mRNA purification, and cDNA synthesis, were performed following previously described protocols [6]. The *E. histolytica ETMP30* gene (EHI_172170) was PCR-amplified from cDNA using Phusion DNA polymerase (New England Biolabs, Ipswich, MA, USA) using the appropriate primer pair listed in Table S1. After restriction digestion with BglII, amplified fragments were ligated into the expression vector containing the hemagglutinin (HA) tag, pEhEx- HA [26] using Ligation-Convenience Kit (Nippongene, Tokyo, Japan), to produce pEhEx-ETMP30-HA. For gene silencing of *ETMP30*, a plasmid was constructed by inserting a 420-bp-long 5’ end of the *ETMP30* protein-coding region that was amplified by PCR, using cDNA as template and the primer pair indicated in Table S1. The PCR-amplified DNA fragment was digested with StuI and SacI and ligated into StuI and SacI-double digested silencing vector, pSAP2-Gunma [10], to produce pSAP2-ETMP30. Transfection of *E. histolytica* cl6 strain for overexpression, and G3 strain for gene silencing was performed by lipofection as described previously [7]. Selection was performed by daily replacement of medium supplemented with Geneticin/G418 (Gibco/Life Technologies, Waltham, MA, USA) at increasing concentration, until control cells (transfected without plasmid) failed to survive the antibiotic challenge.

### 2.3. Immunoflourescence Assay

Indirect immunofluorescence assay (IFA) was conducted as previously described [7] with anti-HA monoclonal antibody (clone 11MO, Covance, Princeton, NJ, USA) to detect HA-tagged ETMP30 diluted 1:500 in 0.1% bovine serum albumin in Tris-buffered saline-Tween 20 (BSA-TBST) and anti-adenosine-5’-phosphosulfate kinase (APSK; EHI_179080; a mitosomal matrix enzyme involved in sulfate activation) polyclonal rabbit antiserum [10] diluted 1:300 in 0.1% BSA-TBST to stain mitosomes. Visualization was done using a confocal laser scanning microscope, LSM780 (Carl Zeiss Microscopy, Oberkochen, Germany).

### 2.4. Immunoelectron Microscopy 

Sample preparation was carried out as previously described [6] with anti-HA mouse antibody, and anti-chaperonin 60 (Cpn60; EHI_178570; a chaperone protein and canonical mitochondrial matrix marker) [10] rabbit antiserum. The grids were observed at Tokai Microscopy Inc. (Nagoya, Aichi, Japan), using a transmission electron microscope (JEM-1400 Plus, JEOL Ltd., Tokyo, Japan) at an acceleration voltage of 80 kV. Digital images (2048 × 2048 pixels) were taken with a charge-coupled device (CCD) camera (VELETA, Olympus Soft Imaging Solution GmbH, Münster, Germany).

### 2.5. Percoll-Gradient Fractionation

Trophozoites expressing ETMP30-HA were washed three times with 2% glucose/phosphate buffered saline prior to collection by centrifugation at 500× *g*, for 5 min at 4 °C. After resuspension in lysis buffer (10 mM MOPS-KOH, pH 7.2, 250 mM sucrose, protease inhibitors), the cells were disrupted mechanically by a Dounce homogenizer. Unbroken cells were removed by centrifugation at 5000× *g* for 10 min, and the homogenate was separated by Percoll-gradient fractionation as previously described [7]. Briefly, the amoebic homogenate was overlaid on 30% Percoll in lysis buffer, before centrifugation at 120,000× *g* for 1 h at 4 °C. Fractions of 200 μL each were collected from top to bottom, while 150 μL of the last eight fractions were pooled and placed on top of a 70% Percoll-lysis buffer solution. Then, 15% Percoll-lysis buffer solution was placed on top of the pooled fractions, prior to a second ultracentrifugation step following the same conditions, after which 200 μL fractions were likewise collected from top to bottom. All fractions from both first and second ultracentrifugation steps were run in denaturing sodium dodecyl sulfate-polyacrylamide gel electrophoresis (SDS-PAGE) and then blotted onto nitrocellulose membranes reacted with either 1:1000 diluted anti-HA antibody or 1:1000 diluted anti-Cpn60 antiserum. 

### 2.6. Sodium Carbonate (Na_2_CO_3_) Treatment of Organelle-Enriched Fractions

Na_2_CO_3_ treatment of organelle-enriched fractions was performed as previously described [5,6]. Briefly, homogenates from trophozoites expressing ETMP30-HA and Tom40-HA [5] were centrifuged at 100,000× *g* for 60 min at 4 °C to separate the organelle and cytosolic fractions. The 100,000× *g* organelle fractions were washed in lysis buffer, and were recollected by centrifugation at 100,000× *g* for 60 min at 4 °C. A total of 1 mg of washed organelle fraction was diluted 20 fold with ice-cold 100 mM Na_2_CO_3_ and 150 mM NaCl, and then incubated on ice for 30 min to liberate soluble and peripheral membrane proteins. The soluble organelle proteins were separated from the membrane-integrated proteins by ultracentrifugation at 100,000× *g*, 4 °C for 1 h. The supernate (soluble organelle fraction) was collected while the particulate (membrane) fraction was washed once with ice-cold 100 mM Na_2_CO_3_ and 150 mM NaCl. Finally, the four fractions containing cytosolic, organelle, soluble organelle, and organellar membrane proteins were subjected to denaturing SDS-PAGE, followed by immunoblotting using 1:1000 diluted anti-HA antibody, anti- Cpn60 antiserum, and anti-cysteine synthase 1 (CS1; EHI_171750; an enzyme involved in sulfur-containing amino acid metabolism) [27] antiserum, respectively.

### 2.7. Quantitative Real-Time PCR (qRT-PCR) Analysis

mRNA expression of *ETMP30* by ETMP30gs and pSAP2G mock strain was analyzed by qRT-PCR, with the gene for RNA polymerase II (*RNApolII*; EHI_056690) used as an internal control. cDNA was synthesized from 5 μg of total RNA by SuperScript III First-Strand Synthesis System (Invitrogen, Waltham, MA, USA) following the manufacturer’s protocol. PCR amplification of the 306 bp segment of *ETMP30* and the 204 bp segment of *RNApolII* was carried out by reacting 50 fold diluted cDNA as template, with appropriate primers listed in Table S1, and Fast SYBR Green Master Mix (Applied Biosystems, Foster City, CA, USA), using the StepOne Plus Real-Time PCR System (Applied Biosystems). The PCR cycling conditions used are as follows: heating to 95 °C for 20 s (enzyme activation), and 40 cycles consisting of two stages; heating to 95 °C for 3 s (denaturation), followed by cooling to 60 °C for 30 s (annealing and extension). The data was analyzed using the ΔCt method [28] and the mRNA expression level of *ETMP30* was normalized to that of *RNApolII*.

### 2.8. Immunoprecipitation (IP) of ETMP30-HA by Anti-HA Antibody

Organelle fractions from ETMP30-HA and mock pEhEx-HA control homogenates were prepared and approximately 2 μg of proteins were solubilized in 2% digitonin in IP Buffer containing 50 mM BisTris-HCl, pH 7.2, 50 mM NaCl, 0.001 % Ponceau S, and 10 % w/v glycerol for 30 min at 4 °C. The solubilized fraction was collected by centrifugation at 20,000× *g* for 30 min at 4 °C. Immunoprecipitation was performed as previously described [5]. Bound proteins were eluted overnight and loaded on SDS-PAGE gels, followed by immunoblotting using mouse anti-HA antibody. Silver staining was performed using Silver stain MS kit (Fujifilm Wako Pure Chemical Corporation, Osaka, Japan), according to the manufacturer’s protocol. 

## 3. Results

### 3.1. ETMP30 is Predicted to Be an Entamoeba-Specific Multi-Pass Membrane Protein

**Figure 1:**
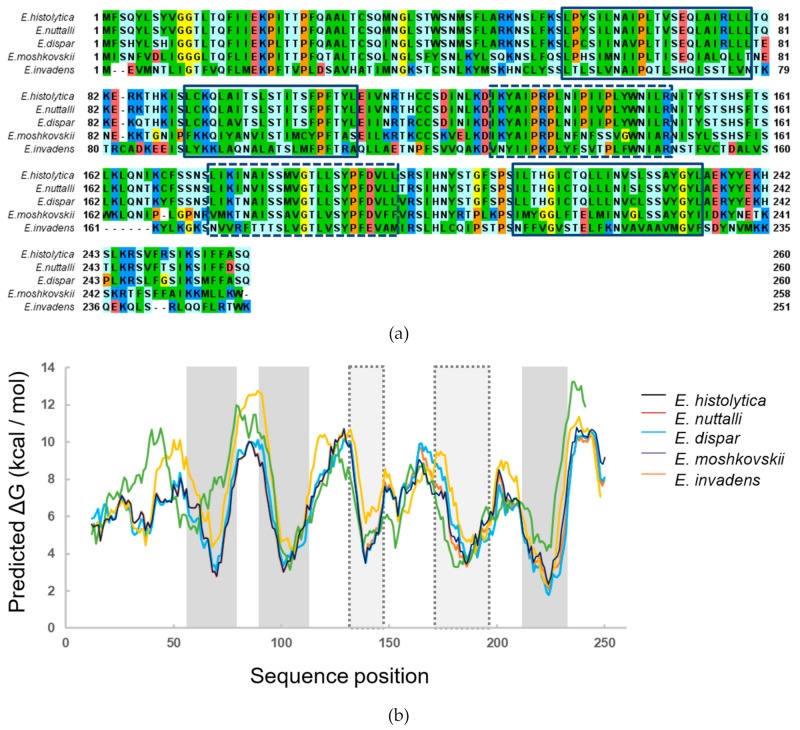
Multiple alignment and transmembrane region prediction of ETMP30 and its *Entamoeba* homologs. (**a**) Multiple alignment of ETMP30 and its *Entamoeba* orthologs was built using MAFFT [29]. The alignment was displayed using Jalview [30]. The hydrophobic, positively charged, negatively charged, hydrophilic, glycine, and proline residues are colored green, blue, red, light blue, yellow, and orange, respectively. Boxes and dashed boxes show predicted transmembrane region of ETMP30 and the region having relatively lower ΔG, respectively. (**b**) Prediction of transmembrane regions of ETMP30 and its ortholog using ΔG prediction for transmembrane insertion [21]. Gray boxes and light gray dashed boxes show predicted transmembrane region of ETMP30 and the region having relatively lower ΔG, respectively.

In our previous work, we developed a prediction pipeline for *Entamoeba*-specific membrane proteins and obtained 25 candidates [14]. Among them, is ETMP30 (EHI_172170), which was initially predicted to be a mitosomal membrane protein having three transmembrane helices. To investigate the conservation of *ETMP30* gene in the *Entamoeba* lineage, we performed sequence similarity search against the proteomes of different *Entamoeba* species available in AmoebaDB [19], using blastp [20]. We detected ETMP30 homologs in *E. dispar* (EDI_154160, with 89% sequence identity and e-value of 8 × 10^−170^), *E. moshkovskii* (EMO_084200, with 58% sequence identity and e-value of 1 × 10^−98^), and *E. invadens* (EIN_036580, with 37% sequence identity and e-value of 1 × 10^−42^), but we could not detect a homolog in the proteome of *E. nuttalli*. We then performed a sequence similarity search against the *E. nuttalli* genome sequence using tblastn [20] and found a very similar sequence in the *E. nuttalli* genome (with 96% sequence identity and e-value of 5 × 10^−172^ Figure 1a), suggesting that the *ETMP30* gene is well conserved in the *Entamoeba* lineage. In addition, we performed further analysis of the transmembrane regions of ETMP30 using ΔG prediction for transmembrane insertion [21], and found that the protein has two additional regions with relatively low ΔG (Figure 1b) in addition to the three predicted transmembrane helices. The five lower ΔG regions were observed not only in *E. histolytica* ETMP30, but also in its homologs, suggesting these proteins likely contain five transmembrane α-helices. 

### 3.2. ETMP30 Is Localized to the Mitosomal Membranes

**Figure 2:**
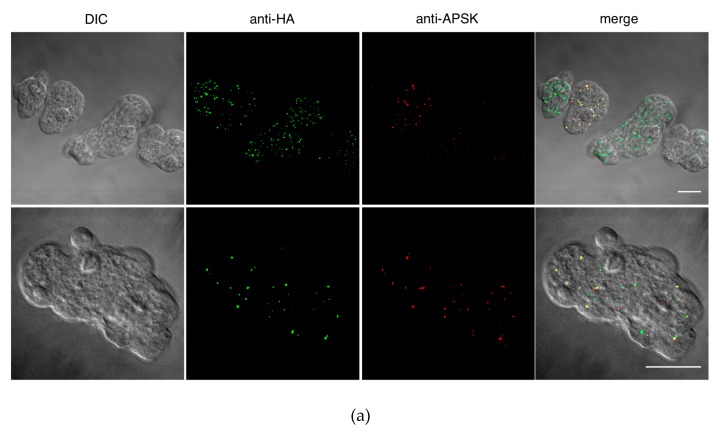

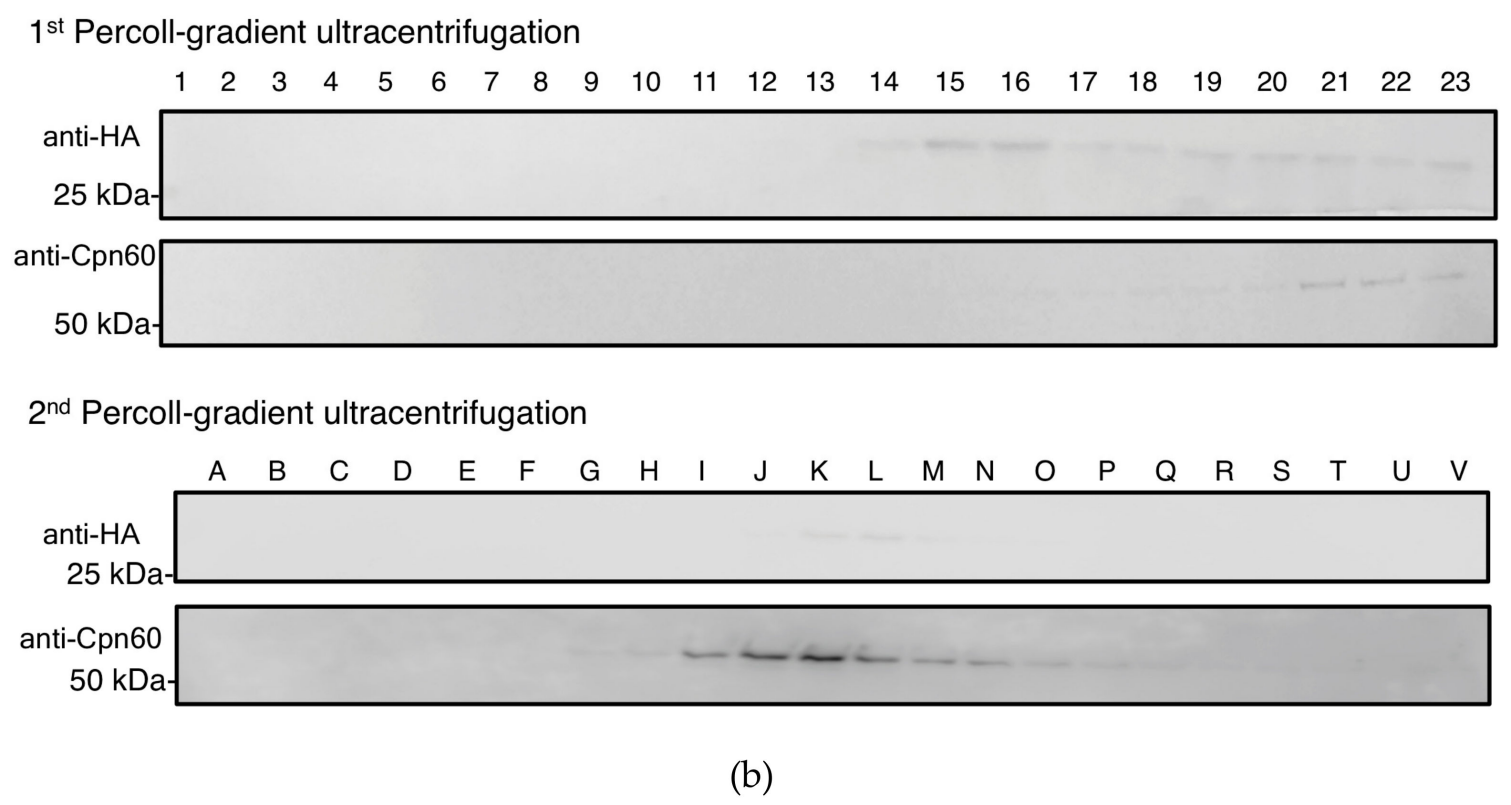
Colocalization of exogenously expressed ETMP30-hemagglutinin (HA) and mitosome marker. (**a**) Immunofluorescence imaging of exogenously expressed ETMP30-HA, stained with anti-HA antibody (green) and the anti-adenosine-5’-phosphosulfate kinase (APSK) antiserum (red). Scale bar, 15 µm. (**b**) Immunoblot analysis after two rounds of Percoll-gradient ultracentrifugation. Approximately 20 μL of each fraction from the first and second ultracentrifugation was separated by sodium dodecyl sulfate-polyacrylamide gel electrophoresis (SDS-PAGE) and transferred to nitrocellulose membranes. The blots were cut into strips containing the region of the target proteins, and then reacted with anti-HA antibodies and anti-chaperonin 60 (Cpn60) antisera respectively.

**Figure 3:**
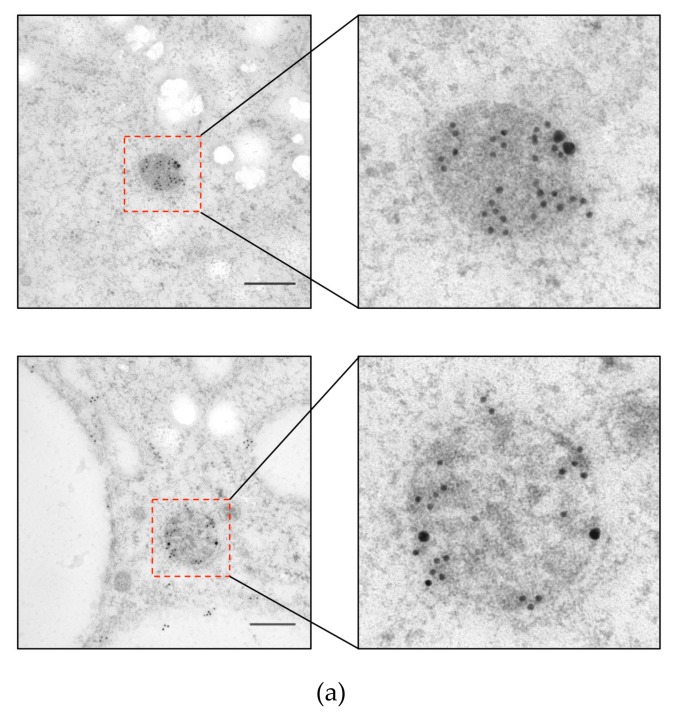

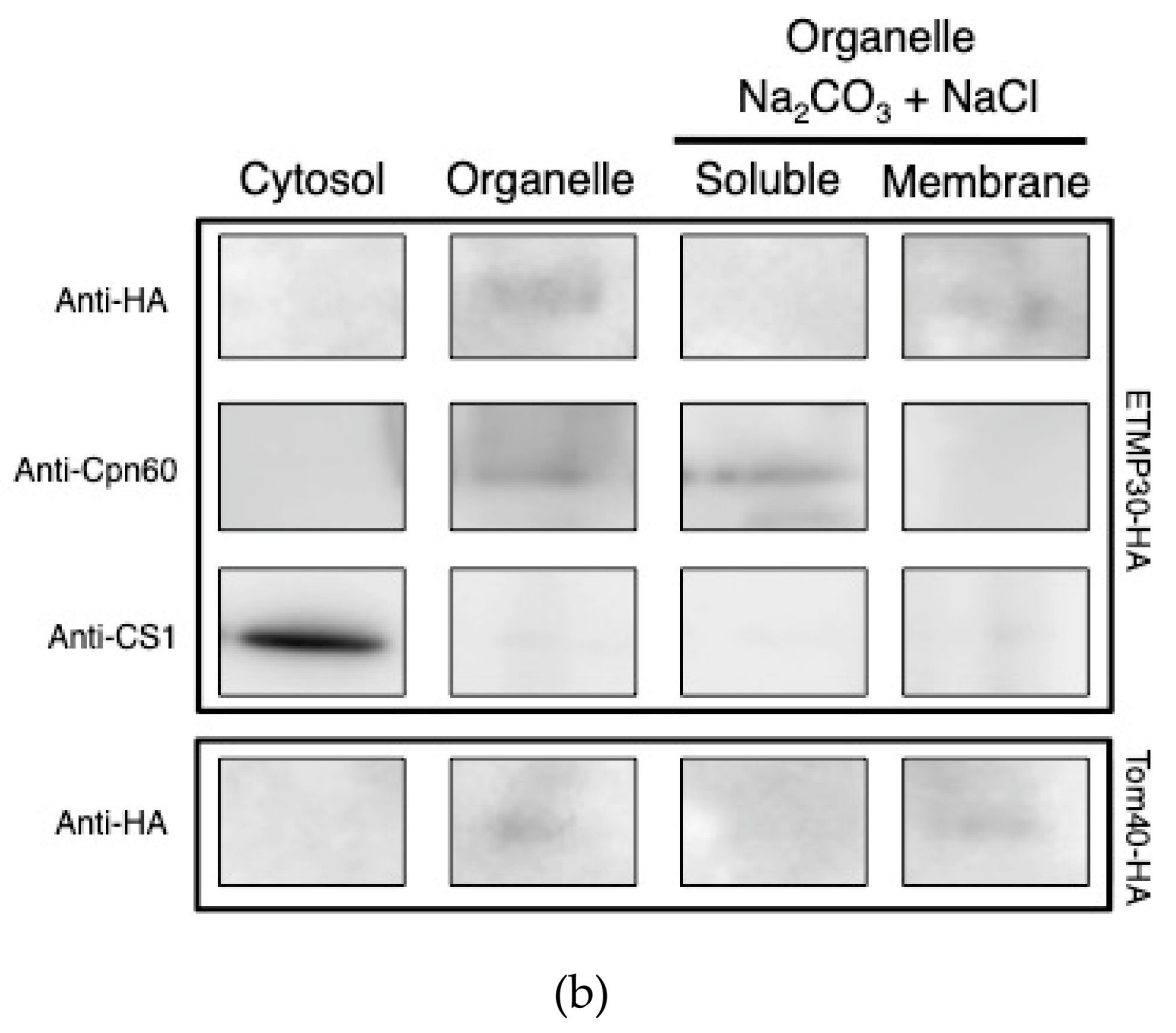
Mitosomal membrane localization of ETMP30-HA. (**a**) Immunodecoration of mitosomes of ETMP30 double antibody staining using anti-HA (15 nm gold particles) and anti-Cpn60 antibodies (5 nm gold particles). Scale bar, 200 nm (**b**) Immunoblot analysis after alkaline carbonate fractionation. Fractions from lysates from ETMP30-HA- (upper box) and HA-Tom40-expressing transformants (lower box; mitosomal membrane protein control) were separated by SDS-PAGE, followed by immunoblotting. Membranes were immunostained with either anti-HA antibody, anti-Cpn60 (mitosomal matrix protein), or anti-cysteine synthase 1 (CS1) (cytosolic protein) antiserum. Relevant portions of the membranes are shown respectively, while source immunoblots are provided in Figure S1.

To investigate the localization of ETMP30 protein, we performed immunofluorescence assay (IFA) using a strain that expressed the candidate protein with a hemagglutinin (HA) epitope tag at the carboxyl terminus, ETMP30-HA, double-stained with anti-HA antibody and anti-adenosine-5’-phosphosulfate kinase (APSK; mitosomal matrix protein) antiserum [10]. IFA results showed punctate and slightly elongated anti-HA signals (Figure 2a). We also observed that some of the anti-HA signals were colocalized with APSK, suggesting the protein is partially localized to mitosomes (Figure 2a). However, some punctate and slightly elongated anti-HA signals did not colocalize with APSK, suggesting that this protein is localized not only to APSK-containing mitosomes but also to other organelles, including a subpopulation of mitosomes where APSK is not present. Independently, we performed fractionation of ETMP30-HA homogenates using Percoll-gradient ultracentrifugation. Immunoblot analysis revealed fractions containing ETMP30 overlapped with those stained with anti-chaperonin 60 (Cpn60; mitosomal matrix chaperon) antiserum [10], suggesting cofractionation of ETMP30 with other mitosome proteins (Figure 2b). Trophozoites expressing ETMP30-HA were also subjected to immunoelectron microscopy analysis to confirm mitosomal membrane localization of the protein by double-staining with anti-HA antibody and anti-Cpn60 antiserum. The electron micrographs of mitosomes from these cells indicated that the protein is localized to the mitosomal membranes (Figure 3a). Moreover, particle distribution analysis of the micrographs of ETMP30-HA expressing trophozoites revealed that the anti-HA antibody-associated gold particle distribution of 49.9 ± 47.6 μm^−2^ in the membranes of mitosomes labeled with anti-Cpn60 antibody was significantly higher compared to 0.27 ± 0.43 μm^−2^ in the cytoplasm (*n* = 10, *p* = 0.009326, using two-tailed Welch’s unequal variance t-test). In support of this observation, we performed carbonate fractionation of ETMP30-HA followed by immunoblot analysis. Our results indicated retention of ETMP30-HA to the organelle membrane fraction even after treatment of the organelle fraction with alkaline carbonate (Figure 3b, upper box, top panel). This pattern is similar to that of Tom40-HA (Figure 3b, lower box), which we used as a positive control for mitosomal membrane protein, suggesting the protein is integrated to organellar membrane. Furthermore, the anti-HA antibody immunoblot profile obtained was different from both the profile of the soluble mitosomal matrix protein, as shown by the blot stained with anti-Cpn60 antiserum (Figure 3b, upper box, middle panel), and that of the cytosolic enzyme involved in sulfur-containing amino acid metabolism, cysteine synthase 1 (CS1) [27], as shown by the blot reacted with anti-CS1 antiserum [27] (Figure 3b, upper box, bottom panel). Overall, these independent imaging and fractionation data strongly indicate that ETMP30 is localized and integrated to the mitosomal membranes.

### 3.3. Transcriptional Gene Silencing of ETMP30 Leads to Growth Retardation and Slight Elongation of Mitosomes

**Figure 4:**
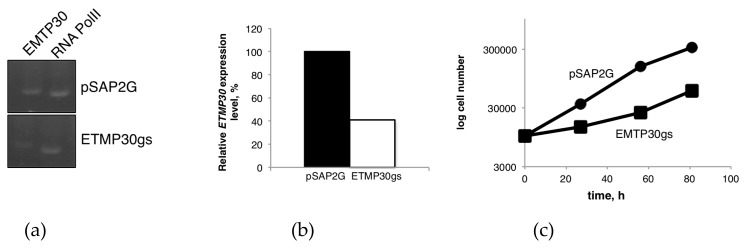

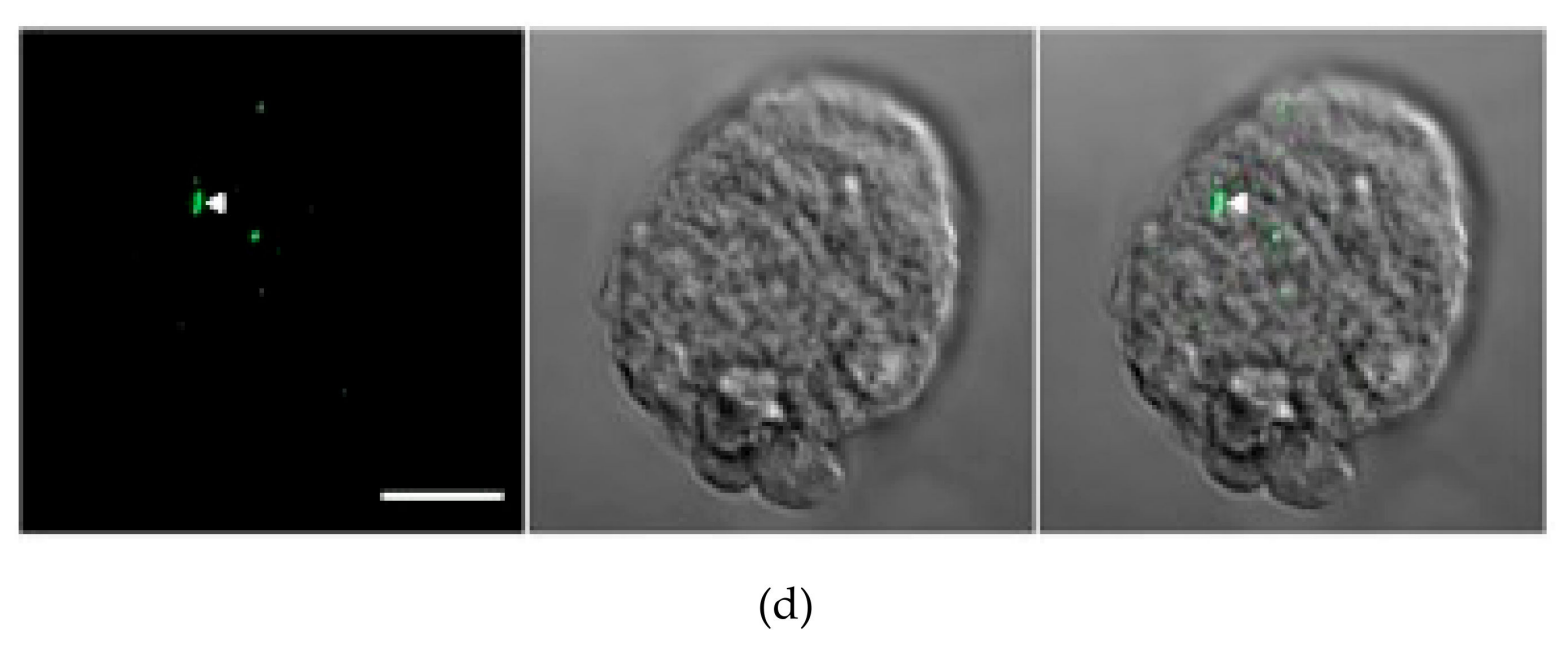
Phenotypic effects of transcriptional gene silencing of *ETMP30*. (**a**) cDNA from total mRNA isolated from ETMP30gs and mock control (transfected with pSAP2-Gunma empty vector) strains, respectively, was used as template. Target segments of the genes were amplified using appropriate primers (Table S1). The 15 kDa subunit of RNA polymerase II gene was used as positive control. (**b**) The specific down-regulation of expression of *ETMP30* mRNA was confirmed by quantitative reverse-transcription PCR of the *ETMP30* gene in ETMP30gs and mock control cells. The amount of *ETMP30* cDNA was normalized relative to that of the gene encoding the 15 kDa subunit of RNA polymerase II. (**c**) Growth curve of ETMP30gs and mock control strains based on cell count performed at different time points within 80 h. (**d**) Immunofluorescence analysis using anti-APSK antiserum. Representative micrograph of ETMP30gs strain with slightly elongated mitosome (white arrowhead) stained with anti-APSK antiserum (green). Scale bar, 5 µm.

We employed transcriptional gene silencing strategy in order to evaluate the biological importance and essentiality of ETMP30. The gene expression of *ETMP30* in both *ETMP30* gene silenced (ETMP30gs) strain and mock control strain transfected with empty vector pSAP2-Gunma was assessed using qRT-PCR. The amount of *ETMP30* transcript was normalized against that of RNA polymerase II. *ETMP30* expression was repressed by 60% in ETMP30gs strain compared to control (Figure 4a,b). We also monitored the growth, and measured the doubling time of both strains. Growth was significantly hampered as a consequence of *ETMP30* gene silencing (Figure 4c). This was also reflected by a two-fold increase in doubling time of ETMP30gs strain compared with the mock control strain (*n* = 3, *p* = 0.0012 using Student’s t-test). We also noticed slight elongation of mitosomes stained by anti-APSK antiserum in the ETMP30gs strain (Figure 4d). However, this phenotype was not as severe as the morphological defects observed in *DrpA* and *DrpB* gene silenced strains and the strains expressing dominant negative DrpA or DrpB mutant reported previously [31].

### 3.4. ETMP30 Interacts with a Cation-Transporting P-Type ATPase

**Figure 5:**
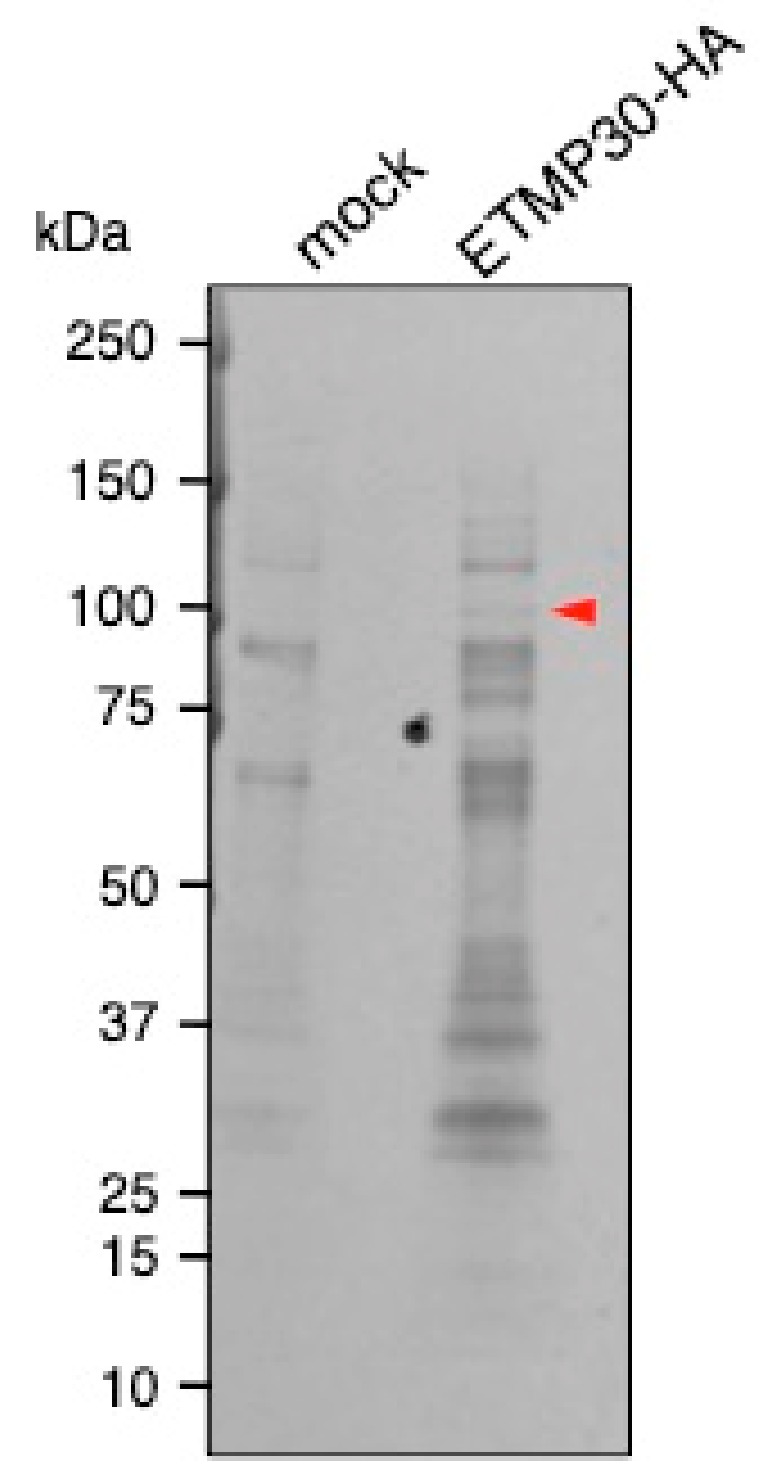
Identification of ETMP30-HA binding proteins from amoebic lysates by immunoprecipitation using anti-HA antibody. ETMP30-HA binding proteins co-immunoprecipitated with anti-HA antibody were separated on SDS-PAGE and detected with silver staining. A red arrowhead indicates the band corresponding to the unique interacting protein of ETMP30. The figure presented is part of the whole gel image shown in Figure S2.

We investigated the potential role of ETMP30 by identifying interacting proteins through immunoprecipitation. Silver-stained polyacrylamide gel detected a band of approximately 100 kDa, which was only present in the sample immunoprecipitated when lysates from the strain expressing ETMP30-HA were used, but absent in the sample from mock control (Figure 5, arrowhead, Figure S2). This specific band was excised from the gel and analyzed by liquid chromatography-tandem mass time-of-flight spectrometry. Identified proteins that range in size from 90 to 130 kDa, and have more than two peptides detected in ETMP30-HA and none in mock control samples are listed in Table 1.

The candidate protein with the highest number of detected peptides, and unique to ETMP30-HA expressing cells was EHI_065670, annotated as cation-transporting P-type ATPase [32]. It shows homology with the human cation-transporting ATPase 13A2 (NP_071372, with 28% identity and e-value of 1 × 10^−79^), that is known as a causative gene for Kufor–Rakeb syndrome caused by lysosomal dysfunction [33]. Recently, this protein was annotated as a member of the secretory pathway calcium ATPases (SPCA) family as it exhibited close phylogenetic relationship to this protein family, demonstrated localization in vacuoles stained with NBD C6-ceramide, a fluorescent dye associated to the Golgi apparatus and subsequently named *Eh*SPCA [16]. The other candidates, EHI_051060 and EHI_030830, showed homology to pyruvate:ferredoxin oxidoreductase and plasma membrane calcium-transporting ATPase, respectively, but both have lower peptide counts detected from the protein sequencing analysis. 

## 4. Discussion

To shed light on other processes involving mitosomes such as quality control, organelle dynamics, biogenesis, and inter-organellar interactions, identification and functional analyses of uncharacterized proteins, including *Entamoeba*-specific proteins, are required. More importantly, the functional identification of unique mitosomal membrane proteins is crucial since they could perform essential roles in this organelle including transport of proteins and metabolites, as well as the establishment of molecular tethers and contact sites with other organelles. Protein and metabolite transport to *Entamoeba* mitosomes remain enigmatic, as only a few membrane protein homologs have been detected in this parasite. This suggests that majority of the transport machineries on the *Entamoeba* mitosomal membranes may have been modified by unique and possibly lineage-specific components. Indeed, most of the identified mitosomal proteins are estimated as gained after divergence to *Entamoeba* [34]. Proof to this was the discovery of a novel *Entamoeba*-specific shuttle receptor Tom60, which is involved in the import of mitosomal proteins from the cytoplasm, via the Tom40 channel [5]. *Eh*MBOMP30, which is likewise *Entamoeba*-specific, was postulated to be a channel protein although experimental data to prove its function remains lacking [6]. Here, we discovered another lineage-specific membrane protein ETMP30, which is predicted to be a multi-pass transmembrane protein. Functional prediction of *Entamoeba*-specific membrane proteins such as ETMP30 is difficult since functional annotated homologous proteins are not found in other eukaryotic species. HHpred [22] predicted weak structural similarity between bovine mitochondrial ADP/ATP carrier protein (Protein Data Bank accession number: 1OKC_A) and ETMP30 with an e-value of 0.024. The mitochondrial carrier family (MCF) proteins exhibit a tripartite structure consisting of three tandemly-repeated domains containing two transmembrane segments separated by extensive hydrophilic regions and a signature sequence motif, PX(D/E)XX(K/R) and (D/E)GXXXX(W/Y/F)(K/R)G [35]. PX(D/E)XX(R/K) is located at the C-terminus of the odd-numbered transmembrane α-helices, whereas (D/E)GXXXX(W/Y/F)(R/K)G is located at the N-terminus of the even-numbered transmembrane α-helices. The three repeats form a structure with a total of six transmembrane α-helices. We searched the repeat sequences in the ETMP30 sequence using HHrepID [23]. However, repeat sequences were not detected. Furthermore, sequence segments matching with the two specific motifs were not found. ETMP30 may be a structural analog of MCFs, but is not likely to have functional similarity with MCF proteins. 

To obtain some clues on the role of this protein, we performed transcriptional gene silencing of the *ETMP30* gene. Results showed that parasites with a reduced *ETMP30* mRNA expression level of 40% (Figure 4b) had severe growth defects (Figure 4c) compared to the control, suggesting the protein may be involved in important but not essential roles in the cell. Furthermore, the slight elongation of mitosomes in ETMP30gs strain (Figure 4d) suggests that this protein may be directly or indirectly involved in mitosomal dynamics. It may also indicate that ETMP30-lacking mitosomes may have impaired functions resulting to the inefficiency of mitosomal fission as reflected by the observed slightly elongated mitosomes in the ETMP30gs strain. 

The potential interaction of ETMP30 with the Golgi-localized *Eh*SPCA, poses an interesting possibility of mitosome to Golgi contact in *E. histolytica* mediated by those two proteins. Although the morphology of the amoebic endomembrane compartments (e.g. ER, Golgi apparatus, Trans-Golgi network) remain unclear [36], several ER-associated proteins such as the ER lumen chaperone, binding immunoglobin protein (BiP) [37,38] and the Ca^2+^-binding protein, calreticulin [39], and Golgi-associated proteins such as N-ethylmaleimide-sensitive factor (NSF) [40], involved in membrane fusion, have been reported. Moreover, a proteomic analysis of the endomembrane system of *E. histolytica* revealed the presence of other ER- and Golgi-associated proteins [36,41], suggestive of the presence of a functional, yet non-canonical endomembrane network in this parasite. 

Multiple alignment of ETMP30 and its homologs shows that most of the putative transmembrane regions have conserved proline. Proline often causes kinks of transmembrane helices allowing them to be flexible and carry out crucial functional roles, for example in G-protein coupled receptors [42]. Those proline residues may likewise regulate the function of ETMP30 and its interaction with *Eh*SPCA. Previously, we reported EHI_099350, a similar lineage-specific mitosomal membrane protein, which has dual localization in mitosomes and ER [14]. In *Giardia*, three lineage-specific mitosomal outer membrane Tom40 interacting proteins (MOMTiPs), namely MOMTiP-5,-7, and -8, were also reported to be localized to both mitosomes and ER [43]. These findings suggest the possible existence of functional homologs of the components that form the ER-mitosome tethering complex analogous to the yeast-specific ER-mitochondria encounter site (ERMES) complex that are yet to be identified [3]. Our finding of ETMP30-SPCA interaction hints at a potential tethering complex that allows interaction between mitosomes and Golgi-like vacuolar compartments. Golgi-mitochondrion contact sites have been reported in pancreatic acinar cells, which is potentially implicated for communication between the two organelles [44]. The close and stable interaction between the two compartments allow an efficient supply of Ca^2+^ and ATP from the adjacent peri-granular mitochondria to the Golgi apparatus [44]. However, one should note that the mitosomes of *Entamoeba* are incapable of producing ATP and the transport and metabolisms of calcium ions have not been well understood in *Entamoeba*. The Golgi apparatus, on the other hand, is also morphologically undefined, although recent reports suggested the presence of Golgi-associated protein homologs and their homologous functions. It remains as an open question what kind of biological processes require such contact between two atypical organelles in *Entamoeba*. We speculate that such contact site may involve cation exchange between the two compartments, which requires further experimental confirmation. Overall, our current approach to look for other lineage-specific proteins with transmembrane domains in the proteomes of parasitic organisms possessing MROs, provide us a springboard to further explore and expand our current understanding of *Entamoeba* mitosomes, that would ultimately provide us a clearer understanding of parasite cell biology and evolution. 

## Figures and Tables

**Table 1:** List of proteins detected exclusively from anti-HA immunoprecipitation of ETMP30-HA after mass spectrometry sequencing analysis. A mock transformant transfected with the empty vector, pEhEx-HA, was used as control.

Annotation	Gene ID	Molecular Weight (kDa)	Coverage (%)	Peptide Number (ETMP30-HA/mock)
Cation-transporting P-type ATPase	EHI_065670	126	9	8/0
Pyruvate:ferredoxin oxidoreductase	EHI_051060	128	3	3/0
Plasma membrane calcium-transporting ATPase	EHI_030830	114	2	2/0

## Supplementary Materials

The following are available online at https://www.mdpi.com/2073-4425/10/5/367/s1, Figure S1: Full-length immunoblots of sodium carbonate fractionation; Figure S2: Full-length silver-stained polyacrylamide gel after anti-HA immunoprecipitation; Table S1: List of primer sets used in this study.
