# Supplementary material for: An Entamoeba-Specific Mitosomal Membrane Protein with Potential Association to the Golgi Apparatus
Source: Genes (Basel). 2019 May 13;10(5):367. doi: 10.3390/genes10050367 (PMC6563013; doi:10.3390/genes10050367)
Supplement: Supplementary file 1 [file genes-10-00367-s001.pdf]

Supplementary Information to

An *Entamoeba*-specific mitosomal membrane protein with potential association to the Golgi apparatus

Herbert J. Santos[1,2,3], Yuki Hanadate[2,3], Kenichiro Imai[4,5], and Tomoyoshi Nozaki[1,2,3,*]

[1] Department of Biomedical Chemistry, Graduate School of Medicine, The University of Tokyo, 7-3-1 Hongo, Bunkyo-ku, Tokyo 113-0033, Japan; hjsantos@m-utokyo.ac.jp , nozaki@m-utokyo.ac.jp
[2] Department of Parasitology, National Institute of Infectious Diseases, 1-23-1 Toyama, Shinjuku-ku, Tokyo 162-8640, Japan; yuki@nih.go.jp
[3] Graduate School of Life and Environmental Sciences, University of Tsukuba, 1-1-1 Tennodai, Tsukuba, Ibaraki 305-8572, Japan
[4] Molecular Profiling Research Center for Drug Discovery, National Institute of Advanced Industrial Science and Technology (AIST), 2-4-7 Aomi, Koto-ku, Tokyo 135-0064, Japan; kenichiro.imai@aist.go.jp
[5] Biotechnology Research Institute for Drug Discovery, National Institute of Advanced Industrial Science and Technology (AIST), 2-4-7 Aomi, Koto-ku, Tokyo 135-0064, Japan

* Correspondence: nozaki@m-utokyo.ac.jp

Received: 9 March 2019; Accepted: 10 May 2019; Published: 13 May 2019

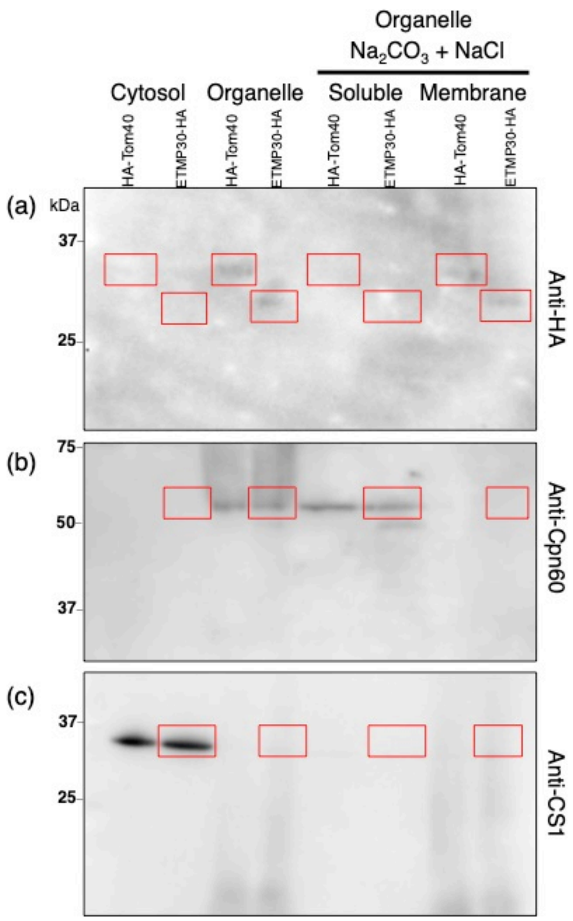

Figure S1. Full-length immunoblots of sodium carbonate fractionation. Fractions from Tom40-HA- and ETMP30-HA-expressing cells were analyzed by SDS-PAGE followed by western blotting. PVDF membranes were reacted with (A) anti-HA antibody, (B) anti-Cpn60, and (C) anti-CS1 antiserum, respectively. Red crop boxes show the regions presented in Figure 3B.

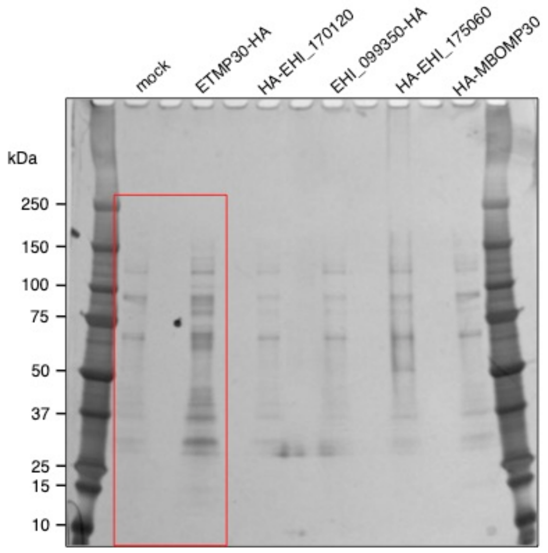

Figure S2. Full-length silver-stained polyacrylamide gel after anti-HA immunoprecipitation. Eluted fractions from the immunoprecipitation of the lysates from ETMP30-HA and mock transformants, were subjected to SDS-polyacrylamide gel electrophoresis followed by silver staining. The red crop box indicates the region presented in Figure 5.

Table S1. List of primer sets used in this study.

For ETMP30-HA expression	
ETMP30-HA_BglII1F	GTTAGATCTATGTTTTCACAGTACCTCAGT
ETMP30-HA_BglIIR	GAAAGATCTCTGAGAAGCAAAAAATATAGAC
For transcriptional gene silencing	
ETMP30gs-StuI-1F	GTTAGGCCTATGTTTTCACAGTACCTCAG
ETMP30gs-SacI-420R	GAAGAGCTCGAGGAATAGCGTATTTAATATC
For confirmation of gene silencing	
ETMP30gs_441F	TTCCTCGACCACTTAATATTCC
ETMP30gs_660R	AATAGCCATAGGCTGATGACAAA
RNApolII_F	GATCCAACATATCCTAAAACAACA
RNApolII_R	TCAATTATTTTCTGACCCGTCTTC
